# Population change and the burden of hospitalization in Germany 2000–2040: Decomposition analysis and projection

**DOI:** 10.1371/journal.pone.0243322

**Published:** 2020-12-11

**Authors:** Enno Nowossadeck, Franziska Prütz, Andrea Teti

**Affiliations:** 1 Unit of Social Determinants of Health, Department of Epidemiology and Health Monitoring, Robert Koch Institute, Berlin, Germany; 2 Unit of Health Reporting, Department of Epidemiology and Health Monitoring, Robert Koch Institute, Berlin, Germany; 3 Institute for Gerontology, University of Vechta, Vechta, Germany; Sciensano, BELGIUM

## Abstract

Demographic factors, such as population aging and shrinkage, and non-demographic factors, such as hospitalization rate and length of hospital stay, generate challenges for inpatient care. This paper used decomposition analysis to assess how changes in these factors affected the number of hospital treatment days from 2000 to 2015 in Germany. Demographic aging was linked to increases in the number of treatment days for women (+10.0%) and men (+19.2%) and in hospitalization rates for women +6.0% and men +5.4%. However, these increases were offset by decreases in the number of hospital days (women: 16.5%; men: 7.3%) and length of stay (women: -27.4%; men -26.3%). For the projection up to 2040, 12 scenarios were developed (six for women and six for men) using three variants for future population demographics and two variants for future length of stay and hospitalization rates. One of the two variants for future length of stay and hospitalization rates provides for a constant value for the year 2015. For the second of these two variants variant, a logarithmic model was estimated on the basis of values from 2000 to 2015. and the trends were extrapolated using this model until 2040. The strongest overall predicted increase was 18.4% between 2015 and 2040, including a 22.4% increase for men. In two scenarios for women, only slight declines were predicted. All results, both for the decomposition analysis and projection, indicated a moderate but sustained effect of demographic aging on the number of hospital treatment days, leading to a significant increase in hospital treatment days over the study period. Non-demographic factors also had strong influences, especially in shorter time periods, but these effects offset each other over time. The change in the population size in the period under study had very little effect on the number of hospital treatment days.

## Introduction

Demographic changes can be rapid, long-term, and irreversible [1, 2]. Demographic change in Germany includes demographic ageing and a shrinking population (at least in a number of regions). Both demographic developments are generating growing challenges for inpatient care as an elementary component of health care in Germany.

Many studies have assessed how population aging affects various health care sectors. Most predict that a growing need for medical care will lead to increases in the use of outpatient [3–5], rehabilitative [6, 7], and nursing [8, 9] services. In Germany, demographic changes, such as aging and shrinking populations in several regions, create challenges for inpatient care, which is an elementary component of health care in Germany [10]. This analysis assessed how population aging affects the number of hospital cases in Germany [11–16], and it provides a novel analysis of the change in total population [17].

This contribution extends the analysis of the effects of demographic change on inpatient care in Germany by looking at hospital days rather than hospital stays, which are both central components of hospital costs. Little is known so far about the effects of demographic change and the effect of non-demographic factors on the number of days hospitals are occupied.

In addition to the demographic factors mentioned above, non-demographic factors are also important for the development of the number of hospital treatment days. These are both hospitalization rates as the number of hospital stays per 100,000 inhabitants and the length of hospital stay [17, 18]. The non-demographic factors themselves are influenced by a number of factors. These can be changes in epidemiological trends, structural changes in the health care system and others (see Table 1).

**Table 1:** Compilation of trends underlying the non-demographic factors.

Epidemiology	Declining incidence rates due to success in prevention, health care and rehabilitation followed by changes in the spectrum of diseases
	Improved chances of survival in potentially life-threatening diseases through improved medical care
Structural changes in health care	Introduction of new therapy options
	Structural changes within the inpatient care sector, e.g. hospital closures, mergers, etc
	Patient shifting between the health care sectors e.g. between inpatient and ambulatory or rehabilitative care
	Introduction of new classification systems, e.g. DRG-system
	Further legal changes to the billing of services
	changes in the utilization, e.g. changes in the use of emergency care
Change of non-demographic structural characteristics of the population	socio-structural changes of subsequent birth cohorts

Source: [17]

Analyses of such trends can inform development of future projections. This study therefore analyzed hospital occupancy from 2000–2015 to make projections for 2016–2040. It had two objectives: (1) to examine the influence of demographic and non-demographic factors on the evolution of hospital occupancy days since 2000 and (2) to project the evolution of hospital occupancy days up to 2040.

## Materials and methods

### Data

This study was based exclusively on publicly available data for 2000 to 2015 from three sources: (1) hospital statistics, (2) population statistics, and (3) the 13th coordinated population projection published by the Federal Statistical Office of Germany (Destatis) [19–21]. All three sources were equally gender- and age-stratified and included 19 five-year age groups ranging from zero to over 90 years. Hospital statistics were collected from all acute inpatient hospitals in Germany for which data of all treatments in a calendar year were recorded [19], excluding inpatient rehabilitation facilities and army hospitals. These statistics show information on hospital treatments, in cases and days. The analysis of hospital statistics included the total number of hospital cases. Thus, persons who were treated more than once in one hospital were counted more than once. Cases with a minimum stay of one day were included, whereas hourly cases without an overnight stay in hospital were excluded.

The population statistics were based on the most recent census in Germany in 2011. This information was broken down by age, gender, and other categories for the respective reporting years [20], using annual averages. The 13th coordinated population projection conducted in 2015 was used to make projections for Germany for all years up to 2060 [21]. The included data from the hospital and population statistics are available online at the Federal Health Monitoring Information System (http://www.gbe-bund.de), as are data from the 13th coordinated population projection, though not differentiated by age or gender. Age and gender data were obtained from Destatis (https://www.destatis.de/EN/Themes/Society-Environment/Population/Population-Projection/_node.html) [21]. All data used are available in the S1–S4 Files.

### Methods

Both the analysis of 2000–2015 and the projection up to 2040 used the following basic equation:

$$D_{t}=\sum_{a=1}^{19}D_{\mathrm{at}}=\frac{\sum D_{\mathrm{at}}}{\sum C_{\mathrm{at}}}*\frac{\sum C_{\mathrm{at}}}{\sum P_{\mathrm{at}}}*\frac{\sum P_{\mathrm{at}}}{P_{t}}{*P}_{t}={\mathrm{LS}}_{\mathrm{at}}{*R}_{\mathrm{at}}{*\mathrm{AG}}_{\mathrm{at}}{*P}_{t}$$ (1)

where D_at_ is the number of hospital treatment days in age group *a* (the 19 five-year age groups ranging from 0–4, 5–9, 10–14, and so on through ≥90) and year *t* (the year of analysis, where 0 and 1 denote the beginning and end of the period studied); C_at_ is the number of hospital cases in age group *a* and year *t*; P_at_ is the population in age group *a* and year *t*; and P_t_ is the total population in year *t*. Thus, ${LS}_{at}=\frac{\sum D_{at}}{\sum C_{at}}$ is the number of hospital days divided by the number of hospital cases (length of stay); $R_{at}=\frac{\sum C_{at}}{\sum P_{at}}$ is the number of hospital cases and inhabitants (hospitalization rate); and ${AG}_{at}=\frac{\sum P_{at}}{P_{t}}$ is the share of population in age group *a* in year *t* (age structure). The data were stratified according to gender and the 19 age groups. Thus, the share of an age group in the total population represents the age structure and, over time, the change in age structure and thus demographic aging.

#### Analysis of the period 2000 to 2015

The paper presents the influence of (non-)demographic factors on the development of hospital treatment days, in the development from 2000 to 2015, employing decomposition analysis. Among demographic factors are: (a) Age structure and (b) population development in general. Non-demographic factors are (c) hospitalization rate, (d) hospital admissions per 10,000 population, and (e) average length of stay (days of occupancy per case). Other non-demographic factors such as disease-specific prevalence or incidence cannot be applied here due to the complexity of the decompensation model.

Decomposition analysis was used to examine the influence of demographic factors and non-demographic factors on hospital days. This approach is frequently used to analyze trends by decomposing trend variables into different components [22], though components should not be interpreted as causes but rather as factors that determine the components [23]. The decomposition method originates from the field of economic research and is suitable for analyzing the effects of demographic aging (e.g., [23]) and other areas of health care research [17, 18, 24–26]. For our analysis we used the calculation formula as used in [18].

The change Δ (quotient of the number of hospital treatment days in year *t1* and in year *t0*) in the number of hospital treatment days from *t0* to *t1* is referred to as the index of hospital treatment days and can be calculated as follows:

$$\frac{\sum D_{a1}}{\sum D_{a0}}=\Delta D=\Delta \mathrm{LS}*\Delta R*\Delta \mathrm{AG}*\Delta P$$ (2)

ΔLS refers to the change in the number of hospital cases as a result of changes in length of stay from *t0* to *t1*, with simultaneous stability of the hospitalization rate, age structure, and total population. ΔLS is therefore a subindex. The interpretation of the other sub-indices ΔR, ΔAG, and ΔP is analogous. ΔR is the effect of hospital treatment rates, and ΔLS is the effect of length of stay on the change of the number of hospital days. ΔAG reflects the effect of changes in age structure and thus demographic aging. ΔP is the effect of changes in population numbers.

The subindices that are kept constant in each case represent weighting factors. Thus, in the present case, ΔLS is weighted with the hospitalization rate, age structure, and population number of the base year. For the other sub-indices, the weighting shifts successively from the base year to the end year [18]. A problem arises, however, because results may vary depending on the order of the sub-indices. To address this problem, it is necessary to calculate all possible combinations of the order of the sub-indices and their arithmetic averages [18, 27].

In addition to the two demographic factors ΔAG and ΔP, two non-demographic factors are included. Although various non-demographic factors are not recorded in the hospital diagnostic statistics, they can be considered in their entirety within the framework of a decomposition analyses of length of stay and hospitalization rate (whose effects are quasi age-standardized) and thus serve as surrogates. To do so, a multiplicative decomposition was applied. The decomposition analysis yielded partial indices whose mathematical products form the overall index. For better readability, the indices are converted into percentages. Because we used a multiplicative decomposition, the sub-indices converted in percentage do not add up to the overall index.

Temporal change was analyzed using two approaches. First, a comparative analysis of the years 2000 and 2015 was conducted without accounting for trends within this period. These trends were considered in the second step, which analyzed the changes on an annual basis. These two approaches yielded chains of sub-indices.

#### Projection up to 2040

A component-based model was constructed [28] to predict the development of the number of days until 2040 by estimating the future number of hospital days based on the four components of Eq (1). Here, the values 0 and 1 for *t* indicate the beginning and end of the projection period. Data for the two demographic factors, age structure and total population up to 2040, were taken from the 13th coordinated population projection. For length of stay and hospitalization rate, logarithmic time series models were used to estimate the data for 2000 to 2015, with parameters applied to a curve-fitting extrapolation up to 2040 [29]:

y=a+b*ln(x) (3)

with

y–length of stay respectively hospitalization rate

x–number of year in analyses respectively projection period (1–2000, …, 41–2040)

and

a–constant

b–coefficient

Existing trends were extrapolated into the future using suitable models. The result was a time series up to 2040 for length of stay and hospitalization rate. In the final calculation step, the gender and age group annual values of the two demographic and two non-demographic factors were used in Eq (1) to estimate the number of hospital days for each year of the projection period.

#### Scenarios

To reflect the diversity of possible future outcomes, different scenarios were developed using assumptions about demographic and non-demographic factors [30–32]. For example, the 13th coordinated population projection includes eight variants and three model calculations based on different assumptions about birth rates, gender-specific life expectancies, and net immigration from abroad [21]. Two of these variants and one model calculation were selected for this paper (see Table 2) to estimate the impact of different demographic trends.

**Table 2:** Overview of the selected variants of the 13th coordinated population projection.

Variant/ Model calculation	Birth rate	Average life expectancy f/m Increase to. . . in the year 2060	long-term net migration
Variant 1	1.4 children per woman	88.8/84.8 years (moderate increase)	100,000 as of 2021
Variant 4	1.4 children per woman	90.4/86.7 years (strong increase)	200,000 as of 2021
Model calculation 2	1.4 children per woman	88.8/84.8 years (moderate increase)	300,000 as of 2021

Source: [21]

As shown in the Table 2, variant 1 is the basic variant, and variant 4 envisages a bigger increase in average life expectancy coupled with increased immigration. The increase in mean life expectancy is mainly due to decreased mortality in older age groups [33]. Model calculation 2 differs from variant 1 in that the annual balance of immigration is significantly higher from 2021 onwards: 300,000 persons per year is significantly higher than the average 241,000 persons for 2000 to 2015. However, compared with the assumption in variant 1 (i.e., 100,000 persons), this assumption is probably underestimated.

Using Eq (1), different assumptions for the future development of the mean length of stay and hospitalization rate were also developed. As previously mentioned, only two approaches were developed here. In the first approach, the values for mean length of stay and hospitalization rate were kept constant at the 2015 level. In the second approach, the mean length of stay and hospitalization rate were extrapolated into the future on the basis of the analyzed development, as shown above (see Table 2).

The calculations were repeated several times by combining the two variants and one model calculation of future population development and the status quo or dynamized scenarios for the length of stay and hospitalization rates (see Table 3). Combining the first approach with variants from the 13th Coordinated Population Projection, scenarios S11, S21, and S31 (see Table 3) were created. These scenarios exclusively depict the effects of demographic development and the differences in expected trends and thus represent status quo scenarios, which refer here to the average length of stay and hospitalization rates but not demographic parameters.

**Table 3:** Overview of the scenarios.

	Scenarios non-demographic	factors
Scenarios demographic factors	Approach 1: Constance	Approach 2: Extrapolation
Variant 1	S11	S12
Variant 4	S21	S22
Model calculation 2	S31	S32

Combining the second approach with the demographic variants, the dynamic scenarios S12, S22, and S32 are created. In dynamic scenarios, the non-demographic factors (length of stay and hospitalization rate) were not kept constant, but a change over the projection period was assumed, provided that a constant course could not be derived from past development. A comparison with the respective status quo scenarios illustrated the effect of the assumptions for mean length of stay and hospitalization rate. Twelve scenarios were created (six for women and six for men). The calculations and evaluations were conducted using R 3.4.1 and MS Excel 14.0.

## Results

In 2015, 19.09 million hospital cases (10.04 million for women and 9.05 million for men) were reported in Germany (Table 4), a 16.9% increase over 2000 (11.6% more for women and 22.9% more for men). The number of treatment days was 144.63 million (75.92 million for women and 67.71 million for men). Treatment days decreased 12.3%, compared with 2000, including a 16.5% decrease for women and 7.3% decrease for men (see Tables 4 and 5). The average length of stay was 7.6 days, which increased to 10.1 days in 2000, with no difference between women and men, and 233.7 cases per 1,000 inhabitants (241.9 women and 225.4 men) were treated in hospitals, a 17.6% increase over 2000 (13.4% for women and 22.8% for men).

**Table 4:** Overview of key figures for inpatient care and the population in 2000 and 2015 by gender and age group.

2000															
	Women						Men								
Age group	Cases	Days	LoS	HR	Share age group	Total population	Cases	Days	LoS		HR	Share age group			Total population
	in 1,000	in 1,000	Days per case	Cases per 1,000	in %	in 1000	in 1,000	in 1,000	Days per case		Cases per 1,000	in %			in 1,000
total	8,972.3	90,900.7	10.1	213.3	100.0	42,071.7	7,364.8	74,100.7	10.1		183.6	100.0			40,116.0
under 5	289.9	2,039.5	7.0	151.0	4.6		391.2	2,635.4	6.7		193.1	5.0			
5 to less than 10	137.1	706.5	5.2	67.7	4.8		179.5	1,017.2	5.7		84.1	5.3			
10 to less than 15	178.8	1,178.4	6.6	77.7	5.5		191.6	1,359.0	7.1		79.0	6.1			
15 to under 20	294.2	2,105.0	7.2	130.7	5.4		216.5	1,692.6	7.8		91.3	5.9			
20 to under 25	403.4	2,727.6	6.8	179.4	5.3		215.9	1,957.4	9.1		92.3	5.8			
25 to under 30	527.4	3,589.2	6.8	212.8	5.9		228.8	2,121.9	9.3		88.0	6.5			
30 to less than 35	640.6	4,616.5	7.2	193.8	7.9		328.2	3,135.6	9.6		93.3	8.8			
35 to less than 40	521.6	4,258.7	8.2	149.7	8.3		401.1	3,913.3	9.8		108.5	9.2			
40 to less than 45	422.5	3,872.5	9.2	137.2	7.3		413.9	4,123.4	10.0		128.3	8.0			
45 to less than 50	437.1	4,202.5	9.6	155.1	6.7		442.7	4,436.6	10.0		154.7	7.1			
50 to less than 55	434.5	4,200.9	9.7	182.6	5.7		462.8	4,558.6	9.9		191.6	6.0			
55 to less than 60	543.8	5,458.4	10.0	211.0	6.1		636.8	6,391.7	10.0		247.7	6.4			
60 to under 65	682.1	7,238.2	10.6	237.8	6.8		807.6	8,406.3	10.4		293.5	6.9			
65 to under 70	636.0	7,379.0	11.6	294.4	5.1		725.6	7,949.7	11.0		377.7	4.8			
70 to less than 75	745.9	9,400.0	12.6	362.5	4.9		704.1	8,039.4	11.4		464.1	3.8			
75 to less than 80	861.2	11,586.7	13.5	452.8	4.5		521.1	6,249.3	12.0		552.0	2.4			
80 to less than 85	511.9	6,996.9	13.7	521.9	2.3		247.7	3,061.2	12.4		612.2	1.0			
85 to less than 90	488.7	6,633.0	13.6	585.2	2.0		185.6	2,302.3	12.4		671.9	0.7			
90 and older	215.4	2,709.9	12.6	541.5	0.9		64.1	748.9	11.7		564.7	0.3			
**2015**															
	**Women**						**Men**								
age group	Cases	Days	LoS	HR	Share age group	Total population	Cases	days	LoS	HR			Share AG	Total population	
	in 1,000	in 1,000	Days per case	Cases per 1,000	in %	in 1,000	in 1000	in 1000	Days per case	Cases per 1,000			in %	in 1,000	
total	10,040.3	75,922.6	7.6	241.9	100.0	41,511.8	9,053.9	68,705.7	7.6	225.4			100.0	40,174.8	
under 5	567.3	2,470.3	4.4	328.3	4.2		655.7	2,901.5	4.4	359.9			4.5		
5 to less than 10	97.9	406.1	4.1	57.0	4.1		129.5	656.1	5.1	71.4			4.5		
10 to less than 15	140.4	979.3	7.0	78.0	4.3		142.2	879.9	6.2	74.7			4.7		
15 to under 20	272.1	1,937.5	7.1	137.0	4.8		202.4	1,263.8	6.2	94.5			5.3		
20 to under 25	341.6	2,015.4	5.9	154.6	5.3		221.9	1,539.2	6.9	93.3			5.9		
25 to under 30	514.1	2,774.5	5.4	201.5	6.1		250.9	1,793.7	7.1	92.1			6.8		
30 to less than 35	559.8	3,037.2	5.4	223.1	6.0		261.8	1,952.1	7.5	100.3			6.5		
35 to less than 40	425.5	2,532.8	6.0	177.2	5.8		274.9	2,017.1	7.3	112.0			6.1		
40 to less than 45	350.5	2,428.2	6.9	139.0	6.1		326.9	2,375.6	7.3	127.4			6.4		
45 to less than 50	477.2	3,582.6	7.5	145.9	7.9		499.4	3,658.9	7.3	148.9			8.3		
50 to less than 55	583.6	4,487.6	7.7	170.5	8.2		655.7	4,892.5	7.5	188.3			8.7		
55 to less than 60	589.4	4,578.3	7.8	197.7	7.2		727.5	5,495.8	7.6	245.4			7.4		
60 to under 65	620.8	4,768.1	7.7	233.2	6.4		766.2	5,884.6	7.7	304.6			6.3		
65 to under 70	620.9	4,865.9	7.8	286.5	5.2		736.5	5,791.9	7.9	367.7			5.0		
70 to less than 75	838.7	7,014.0	8.4	373.8	5.4		923.1	7,559.7	8.2	472.5			4.9		
75 to less than 80	1,108.4	9,872.8	8.9	473.9	5.6		1,067.6	9,151.3	8.6	576.9			4.6		
80 to less than 85	870.3	8,255.5	9.5	590.7	3.5		683.3	6,139.4	9.0	692.0			2.5		
85 to less than 90	671.4	6,420.9	9.6	684.9	2.4		390.9	3,551.2	9.1	794.4			1.2		
90 and older	390.4	3,495.6	9.0	714.9	1.3		137.3	1,201.5	8.7	871.2			0.4		

Abbreviations: LoS: length of stay; HR: Hospitalization rates

Source: Hospital statistics 2000–2015 [19], population statistics 2000–2015 [20], own calculations

**Table 5:** Development of the number of treatment days (in %), 2000–2015, results of decomposition analysis.

	women	men
overall development	-16,5	-7,3
thereof		
• change due to length of stay	-27,4	-26,3
• change due to hospitalization rates	6,0	5,4
• change due to ageing	10,0	19,2
• change as a result of population number	-1,3	0,1

Source: Own calculations

**Fig 1:**
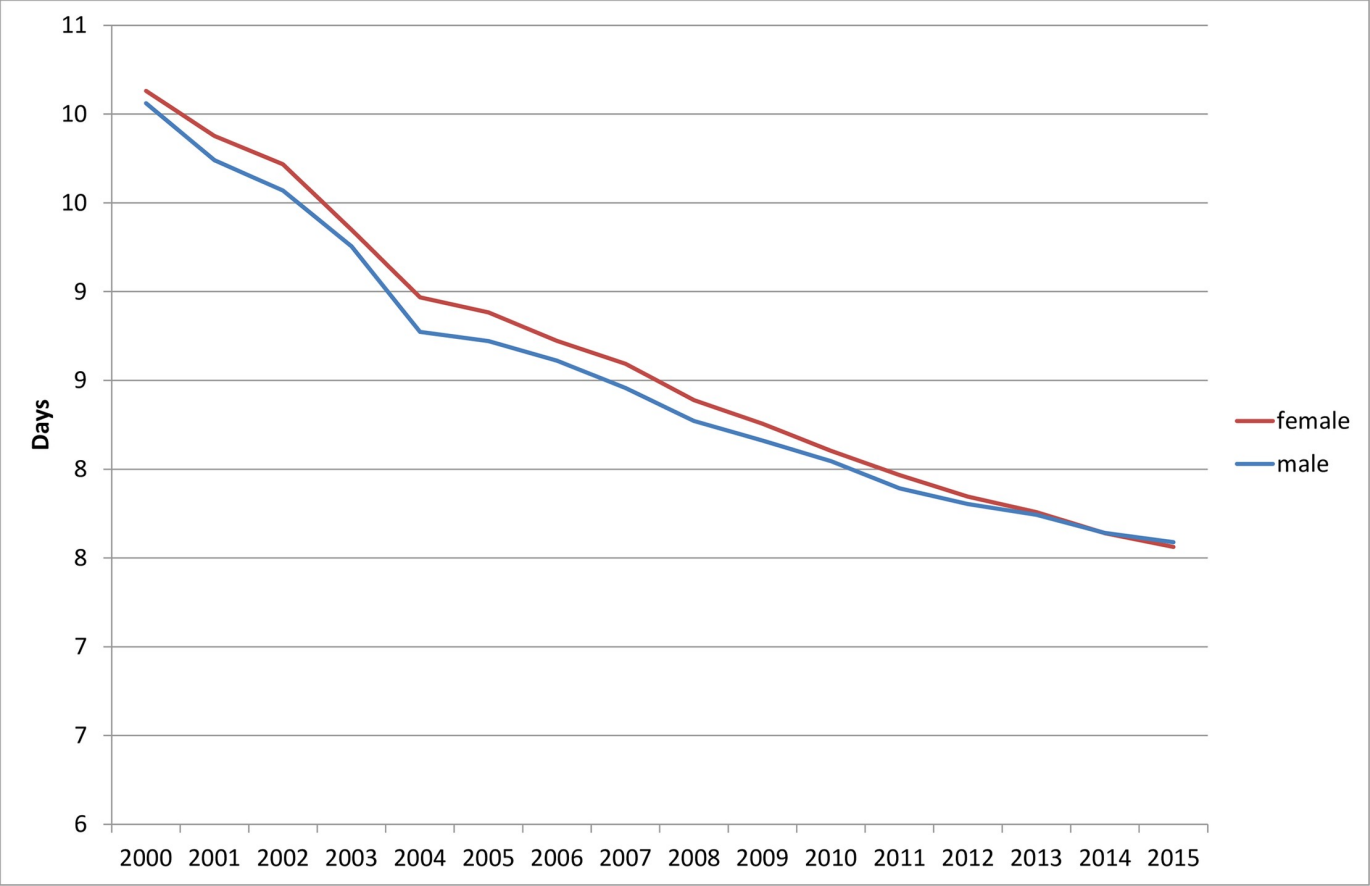
Average length of stay in hospitals (day per case), 2000 to 2015. Source: Hospital statistics 2000–2015 [19].

The average length of hospital stay did not decrease linearly between 2000 and 2015. On the contrary, a decreasing dynamic is discernible (i.e., the average length of stay at the beginning of the period under study decreased more than at the end), as shown in Fig 1. The differences between women and men were minor, even though the range of values on the y-scale was limited to 6–11 days.

**Fig 2:**
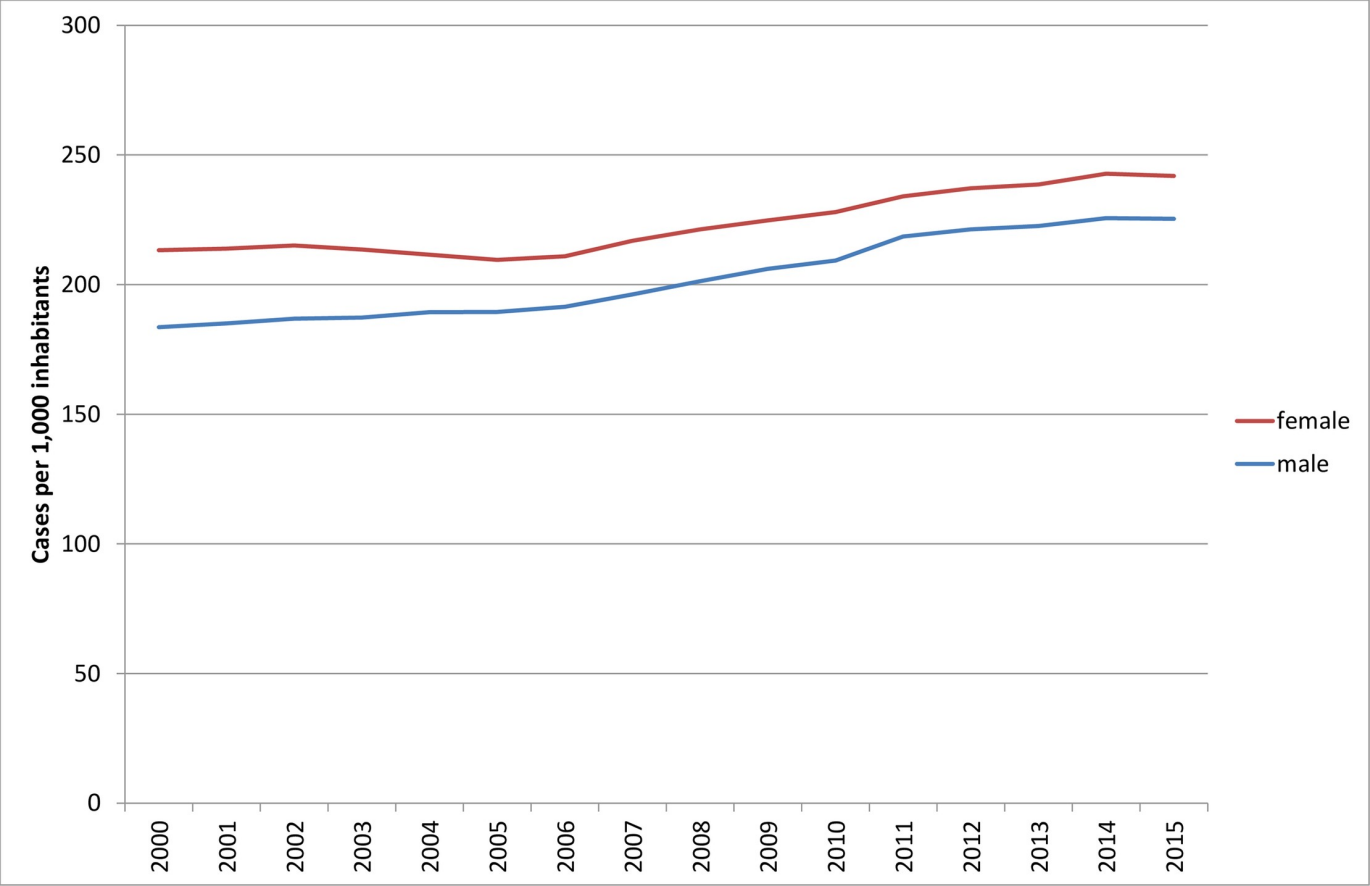
Hospitalization rates (hospital cases per 1,000 inhabitants), 2000 to 2015. Source: Hospital statistics 2000–2015 [19].

The hospitalization rates also were not linear. Rates were constant until the mid-2000s, then began to rise, and then levelled off toward the end of the study period (Fig 2).

### Decomposition of data from 2000 to 2015

The decrease in the number of treatment days was mainly due to changes in the average length of stay between 2000 and 2015. If considering only this development, the number of treatment days would have fallen by more than a quarter (27.4% for women and 26.3% for men), as shown in Table 5. However, when considering demographic aging, the number of treatment days would have increased by 10.0% for women and by 19.2% for men. The increased hospitalization rates resulted in increases of 6.0% for women and 5.4% for men in the number of treatment days. It should be noted that, as shown in the methods section, the percentage sub-indices do not add up to the overall index.

**Fig 3:**
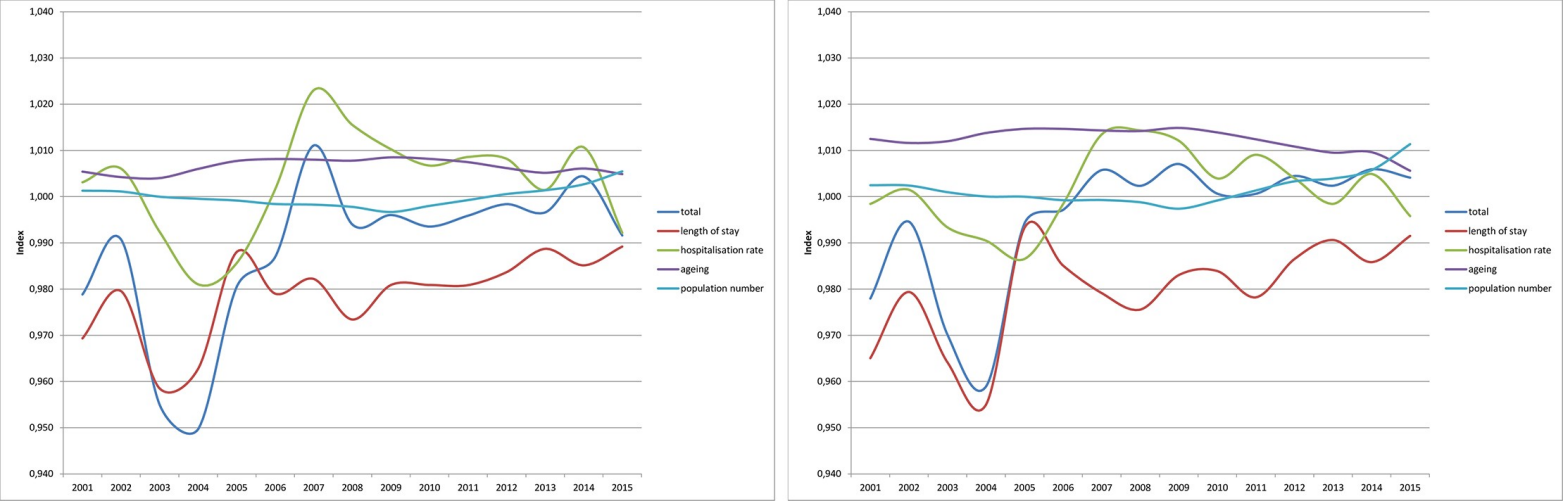
Decomposition of the hospital days 2000–2015: Development of the sub-indices (a) women and (b) men. Source: Hospital statistics 2000–2015 [19], population statistics [20], own calculations.

For the period analyzed, the sub-indices were formed on an annual basis and then linked together. Fig 3 shows the results for the number of treatment days. Values smaller than one indicate decreasing treatment days due to the respective component, compared with the previous year. Values larger than one correspondingly indicate increasing treatment days. For better orientation, a black line on the Y axis denotes the value of one.

### Projection up to 2040

**Fig 4:**
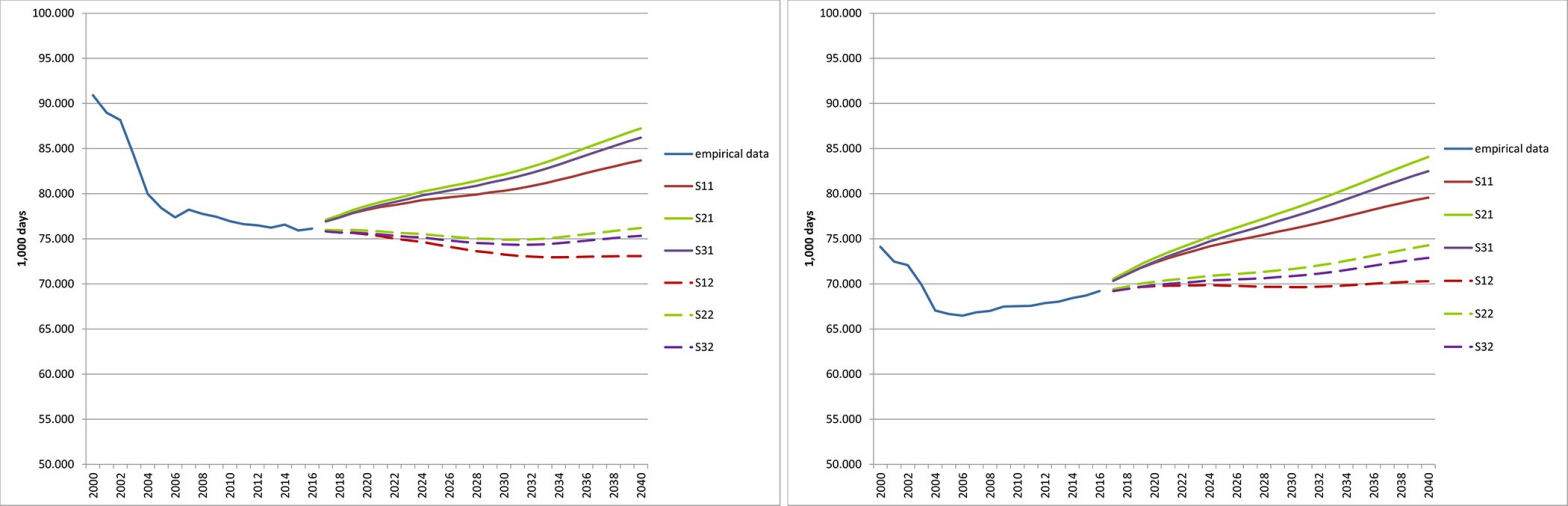
Projection of the number of treatment days until 2040 (in 1,000). (A) women (B) men. Source: Hospital statistics 2000–2015 [19], population statistics [20] own calculations.

Fig 4 and Table 6 show the empirical results of the scenarios predicting the number of treatment days.

**Table 6:** Results of the projection of the scenarios: Treatment days in 1,000, women and men, 2015–2040.

Scenario	Women		Men		Both	
Year	Number of days	Change compared to 2015 (%)	Number of days	Change compared to 2015 (%)	Number of days	Change compared to 2015 (%)
2015	75,923		68,706		144,628	
S11						
2020	78,214	3.0	72,333	5.3	150,547	4.1
2030	80,311	5.8	76,076	10.7	156,387	8.1
2040	83,678	10.2	79,554	15.8	163,233	12.9
S21						
2020	78,648	3.6	72,835	6.0	151,483	4.7
2030	82,125	8.2	78,263	13.9	160,388	10.9
2040	87,222	14.9	84,065	22.4	171,288	18.4
S31						
2020	78,330	3.2	72,446	5.4	150,777	4.3
2030	81,527	7.4	77,390	12.6	158,917	9.9
2040	86,206	13.5	82,489	20.1	168,695	16.6
S12						
2020	75,479	-0.6	69,744	1.5	145,223	0.4
2030	73,247	-3.5	69,643	1.4	142,889	-1.2
2040	73,086	-3.7	70,293	2.3	143,379	-0.9
S22						
2020	75,895	0.0	70,224	2.2	146,119	1.0
2030	74,904	-1.3	71,636	4.3	146,541	1.3
2040	76,198	0.4	74,273	8.1	150,471	4.0
S32						
2020	75,590	-0.4	69,852	1.7	145,442	0.6
2030	74,368	-2.0	70,851	3.1	145,219	0.4
2040	75,329	-0.8	72,884	6.1	148,213	2.5

Source: Hospital statistics 2000–2015 [19], population projection 2016–2040 [21], own calculations

Scenario S21 had the highest increase of 18.4% (women and men) between 2015 and 2040, with the strongest overall increase in this scenario for men at 22.4%. Two scenarios for women showed slight declines: -0.8% in scenario S32 and -3.7% in scenario S12.

#### Gender differences

For women, the status quo scenarios S11, S21, and S31, which were based exclusively on demographic trends, showed an increase of 10.2% to 14.9% in the number of treatment days between 2015 and 2040 (Table 6). The number of treatment days was lowest in scenario S11 (Fig 4A). For better differentiation of the scenarios, the lower value of the y-scale was set to 50,000,000 days in the graphs of Fig 4. In contrast, the dynamized scenarios S12, S22, and S32 showed a slight decline in the number of treatment days until around 2030, continuing a decline that started in the mid-2000s. However, given the length of the projection period, these data are more likely to be constant.

For men, the status quo scenarios S11, S21, and S31 continued to increase from the mid-2000s (see Fig 4B), rising between 15.8% and 22.4% (Table 6). The dynamized scenarios S12, S22, and S32 suggest an initial large constant number of treatment days, then the numbers rise slightly in scenarios S32 and S33 starting around 2030. In general, the number of days for both women and men in the status quo scenarios is higher than the number of dynamic days.

The differences between the purely demographic scenarios S11, S21, and S31 amounted to 4 percentage points for women and 6 percentage points for men in 2040. Scenarios S12, S22, and S32 resulted in differences of 5 percentage points for women and 7 percentage points for men. The differences between the demographic scenarios on the one hand and the dynamic scenarios on the other are to be expected, with approximately 14 percentage points for both women and men.

#### Age differences

**Fig 5:**
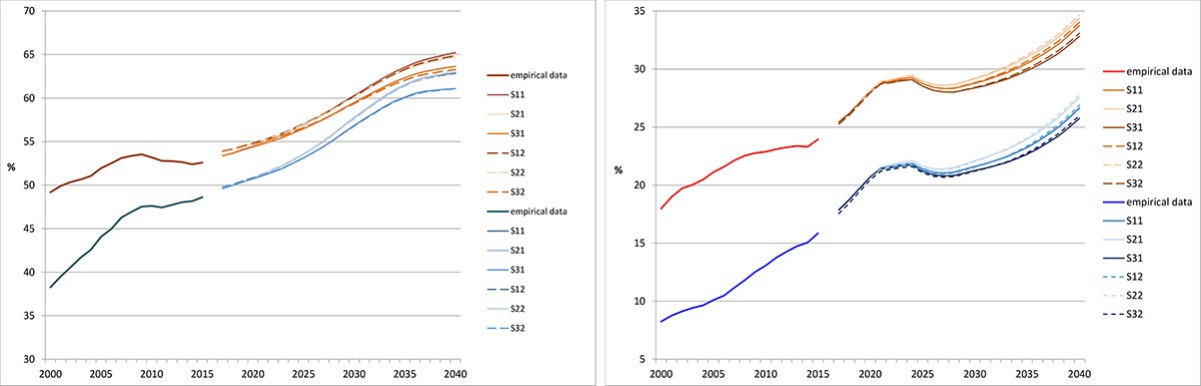
Percentage of treatment days of patients of different age groups on all treatment days. (A) 65 years of age or older. (B) 80 years of age or older. Red and reddish colors: woman; blue and bluish color: men. Source: Hospital statistics 2000–2015 [19], population projection 2016–2040 [21], own calculations.

In 2015, approximately half of all treatment days were devoted to patients aged 65 years or older (52.6% women, 48.6% men). This and the following figures are not presented. At the beginning of the analysis period, the figures were 49.2% for women and 38.8% for men. At the end of the projection period, they were between 63.3% and 65.2% (women) and 61.1% and 63.0% (men), depending on the scenario (Fig 5A). The increase between 2015 and 2040 varied between 18.7% and 42.2% (women) and 32.3% and 58.6% (men), depending on the scenario. In comparison, in 2015, 23.9% of women and 15.9% of men aged 80 or older were treated. This and the following figures are not shown. In 2000, the respective rates were 18.0% for women and 8.2% for men. These percentages were projected to be between 33% for women and 26% for men in 2040, depending on the scenario (Fig 5B). The results indicate growth rates between 36.9% and 65.0% for women and 73.5% and 112.7% for men. The number of treatment days for older people showed a temporary dip between 2024 and 2028, as indicated by the proportion of treatment days for this age group (Fig 5).

**Fig 6:**
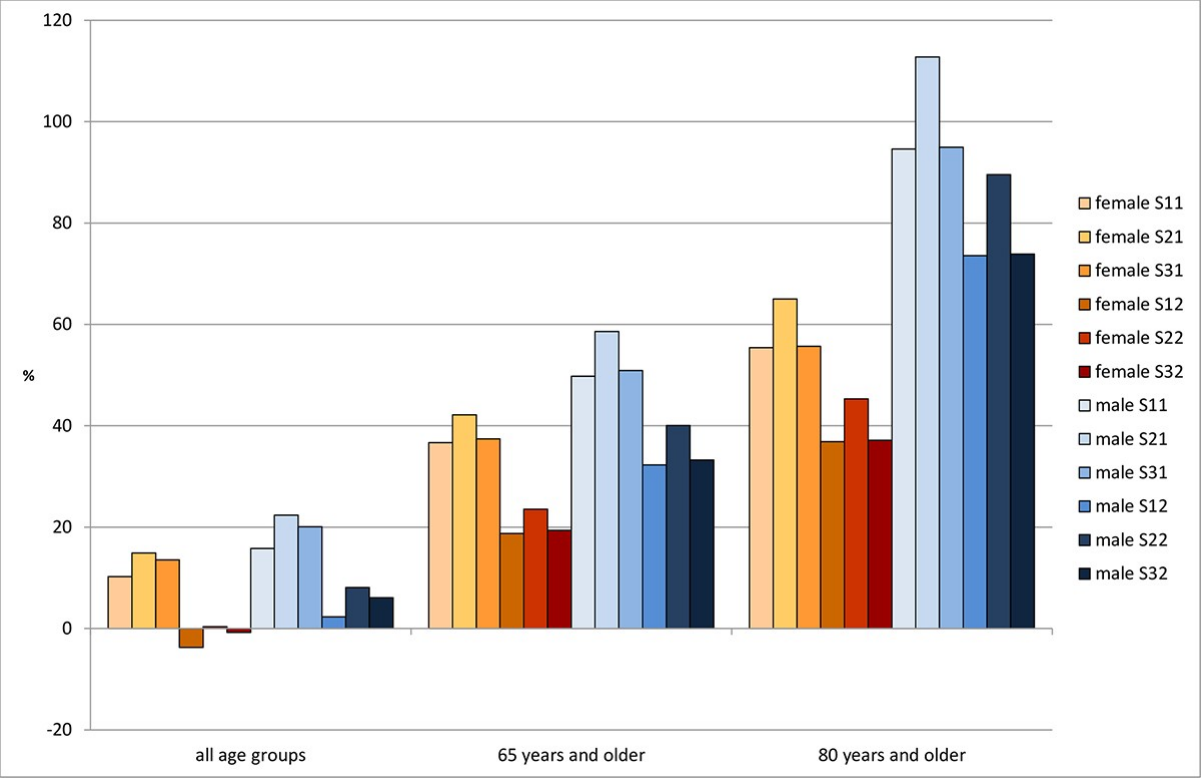
Change in the total number of treatment days, in the age group 65 years of age or older and 80 years of age or older. Source: Population projection 2016–2040 [21], own calculations.

**Fig 7:**
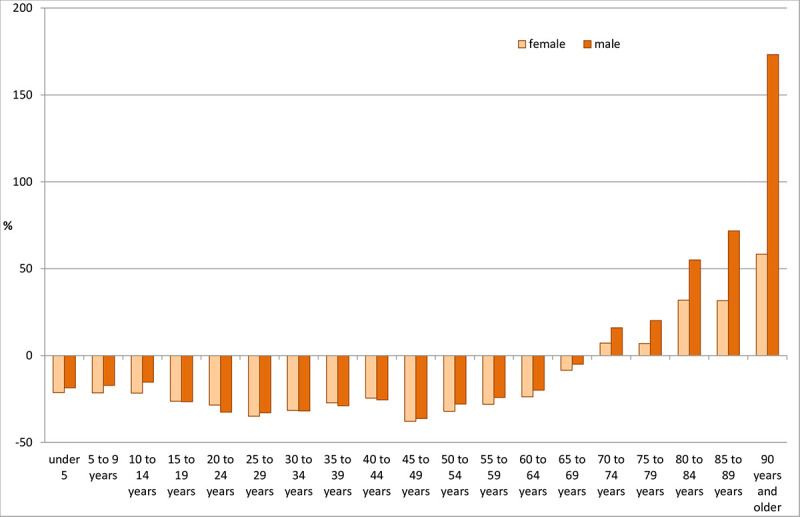
Change in the number of treatment days in 2040 compared to 2015 by age group and sex, scenario S12. Source: Population projection 2016–2040 [21], own calculations.

As Fig 6 shows, the change in the number of treatment days in all scenarios for both women and men in the two age groups (≥65 years and ≥80 years) is greater than in the total population and greater in the ≥80 age group than in the ≥65 age group. Fig 6 shows two scenarios for women in which the total number of treatment days decreases. Scenario S12 showed the strongest decline of -3.7% (Table 6). In the higher age groups, such a decline was not observed, indicating that the decline resulted from age groups other than those ≥65. Accordingly, Fig 7 shows the change in the number of treatment days between 2015 and 2040 by age group. For both women and men, the number of treatment days in all age groups fell below a threshold of 70 years.

## Discussion

The paper analyzed the development of hospital treatment days between 2000 and 2040 in Germany. Development from 2000–2015 was examined by means of a decomposition analysis of the number of treatment days using length of stay, hospitalization rate, age structure, and population size. Projections were made for the period up to 2040.

Demographic and non-demographic factors were included simultaneously in the analysis of the number of hospital days. The annual effects of demographic aging can at best be described as moderate. Non-demographic factors can modify the development in the short to medium term, sometimes very strongly. However, the long-term development, which can also be demographically determined, continues, possibly at a different level (shift).

Another noteworthy result is that the number of hospital cases increased between 2000 and 2015, but the number of treatment days decreased. This result can be explained by the decreasing length of stay, which occurred mainly between 2002 and 2005 and was accompanied by a decrease in the number of hospital cases [17]. As the results of the decompensation analysis show, both declines were strong enough to overcome the effects of population aging. This period was followed by a phase in which hospitalization rates rose and aging continued to have an increasing effect on the number of hospital days.

Since 2011, the number of hospital days has increased, but length of stay has continued to decrease. One reason for this almost indirectly proportional development could be the introduction of Diagnosis Related Groups in Germany in 2004 [34–36]. Since then, hospitals no longer charge daily rates, but instead assess fees on the basis of diagnosis-related groups. Compared to the old system of daily rates, Diagnosis Related Groups provide stronger incentives for economic behavior, to the detriment of patients’ length of stay in hospital [37–39]. However, this structural change due to the shortened lengths of stay may be responsible for an increased need for outpatient follow-up treatment and for increasing complication-related follow-up admissions to hospitals [37, 40–42].

The changes in mean lengths of stay and hospitalization rates 2000 to 2015 show a nonlinear course, so a nonlinear logarithmic function was applied for the extrapolation up to 2040. The results of the federal-level decomposition analysis showed that a population decline slightly alleviated the effects on the number of treatment days [17]. This result may occur again at the federal level if the overall population in the Federal Republic of Germany declines. The mean length of hospital stays had a large effect, indicating that non-demographic factors can strongly influence the number of occupancy days but that the effects of demographic developments are not inevitable.

A gender comparison of the four factors showed that the effects of the two non-demographic factors differed only slightly between women and men, whereas aging in men had almost twice the effect on the number of treatment days (+10% for women and +19.2% for men). This result indicates that changes in the health care system, as reflected by these two non-demographic factors, did not have quantitatively different consequences for men and women. However, aging had greater effects on men than on women, particularly due to two demographic trends. First, in recent years, the life expectancy of men in Germany has risen somewhat more strongly than it has for women, narrowing the existing gap [43]. Consequently, the numbers and proportions of older men increased more than it did among women. Second, the oldest age groups to date have been those most severely affected by war losses during the Second World War, resulting in a gender disproportion. Subsequent generations, which were not affected by this disproportion, are now aging, again leading to increased aging in men [17].

The projections up to 2040 were conducted using 12 scenarios (six for men and six for women), each with three different assumptions about future demographic development (two variants and one model calculation using the 13th coordinated population projection by Destatis) and two different assumptions (status quo and non-linear extrapolation) for the length of hospitalization and hospitalization rate. The results of the various scenarios were conditional (“if-then”) statements: if the underlying assumptions occur, then the future development will proceed according to the results of the respective scenario. This condition also applied to status quo scenarios and projections that consisted of only one scenario.

### Comparison of status quo scenarios

Scenario S11 showed the smallest increase in number of treatment days for both women and men (Fig 4). This scenario assumed an annual migration balance of 100,000 persons (from 2021) and a moderate increase in average life expectancy (compared to scenario S21). Assuming an annual migration of 200,000 persons and a sharp increase in life expectancy (S21), the highest projected numbers of treatment days were obtained. Scenario S31 was based on an annual net migration of 300,000 persons and a moderate increase in life expectancy, and it showed growth slightly lower than in scenario S21. Immigration thus had less strong effects than the increase in life expectancy, at least in the quantitative terms assumed in the scenarios.

Similarly, the increase in life expectancy was the decisive demographic component for the development of the number of treatment days. Immigration did not have a particularly significant effect in this analysis, which makes sense considering the age structure of immigrants. Migrants are on average much younger than the German population, and they are predominantly men [44]. Young men have a comparatively low need for inpatient care. It thus takes more years than the projection period to reach an age at which this need grows substantially.

The scenarios that include an extrapolation of the mean length of hospital stay and hospitalization rates (S12, S22, S32), in addition to demographic trends, appeared to shift downwards, compared with scenarios S11, S21, and S31 (Fig 4). The differences within the two scenario groups S11, S21, S31 and S12, S22, S32 were smaller than those between these two groups. This result means that accounting for different demographic trends led to smaller differences than keeping the non-demographic factors constant over the projection period or making them more dynamic.

### Comparison of the status quo with the dynamic scenarios

Compared with the dynamic scenarios, all status quo scenarios had higher increases in treatment numbers, both for women and men. As previously described, the dynamic scenarios also yielded constant figures, whereas the status quo scenarios showed that demographic aging led to increased treatment days. The two non-demographic factors apparently compensated for the effects of demographic developments up to 2040 for women and mid-2030 for men. However, this compensation effect did not happen automatically and required appropriate efforts. The question arises as to whether future developments could further reduce the average length of hospitalization and hospitalization rate. Such developments could include shifting services to other health care sectors, such as outpatient care [45] and outpatient surgery [46]; changes in medical indications and patient preferences, such as fewer hysterectomies [47, 48] or changing Caesarean section rates [49]; improved therapies and interventions; and epidemiological trends of decreasing incidence and prevalence.

#### Age structure

Compared with the low overall number of treatment days, the dynamics in the different age groups were much more differentiated, indicating that increases were larger in the older age groups than in the population as a whole. Moreover, in scenario S12, the number of treatment days increased exclusively in those aged 65 to 70 years. Differences between the other scenarios were small for those aged 65 years and older. In those aged 80 years and older, a temporary dip was projected for the period between 2024 and 2028. Those born between 1944 and 1948 belong to this age group, which is characterized by very low birth rates. From 2028 onwards, the birth cohorts from 1949 onwards will include successively more births. After 2028, the trend will become much more dynamic again and will probably continue until at least 2040.

One important finding is that the inpatient care of the elderly and especially the very old will gain disproportionately in importance. The focus is not only on the increase in the number of hospital days, but also on related problems such as multimorbidity, polypharmacy, geriatric care etc.

The current pandemic caused by the SARS-CoV2 virus shows two things with regard to stationary care in Germany. First, such an event with presumed or real consequences for inpatient care is not predictable and thus cannot be the subject of a scenario or considered in a scenario. Second, in expectation of a pandemic with many hospital stays, intensive preparatory measures were taken in hospitals in Germany in the first months of 2020. The number of intensive care beds increased, and non-urgent operations were postponed. Necessary treatments also may have been delayed because people were afraid of becoming infected when using the health system. As a result of these measures, the pandemic outcomes in Germany were more favorable than initially feared and more favorable than in other countries (i.e., less severe cases and fewer reported deaths). Altogether, there may even have been fewer hospital stays in the first half of 2020 than in previous years, especially in less affected regions, and fewer hospital stays than in our projection estimates. The impact of a possible second wave is not foreseeable at present, especially if it occurs before the widespread availability of efficient drugs or vaccines. However, given the health system and the experience gained, there is hope that a second wave could be dealt with in a similarly favorable way. In this respect, the COVID-19 pandemic may have no substantial effects on the development of future case numbers in inpatient care in Germany.

## Conclusion

Demographic aging had an increasing effect on hospital case numbers and days, but these effects were not "explosive." Rather, they happened gradually from year to year, but they were permanent. This finding is not new (e.g. [17]). Such trends have been observed since 2000, and similar ones are projected up to 2040. Still, sudden and undetected shifts in demographic factors can have severe consequences for health care, as postulated by the media and some scientific articles (e.g., [50–52]), particularly in connection with the number of treatment days. Moreover, the consequences can persist year after year. Whether they are actually perceived by the relevant actors is another question. The use of predictive formulations and models can increase the chances of recognizing these changes early enough to implement adequate countermeasures.

It should also be noted that the phrase "time bomb" implies that a future increase in hospital treatment days is a problem. However, the expected outcome of hospital treatments is a positive one: a gain in lifetime and quality of life. In this respect, an increase in hospital treatments can be positively connotated as well, and it is a societal or political decision in what amount of resources should be spent on it.

The sustainable impact of demographic change does not require one-off interventions, which then "only" have a one-off effect, but rather measures whose impact is permanent. These include prevention measures, namely primary, secondary and tertiary prevention.

In principle, epidemiological, medical, and therapeutic technology will continue. Such innovation may offer treatment for diseases that were poorly understood or not treatable (e.g., a communicable disease outbreak with severe progression, high transmission rates, and difficult-to-control transmission pathways), which drive up hospitalization rates and lengths of stay in the short term. Such options are beyond any non-speculative prediction, however, and therefore played no role in the calculations presented here. In analyses where it is impossible to predict sudden breaks in trends in major proportions, including their direction and pace, a method that extrapolates trends from the past via the present into the future can be applied [53]. These extrapolations can minimize the uncertainty associated with future trends.

The analyses outlined here found no explosive effects. This finding must not be taken as an indication to do nothing, however. Even if future problems are not related to total number of treatment days, the demographic shifts in population age had important effects in all scenarios. The increased number of treatment days among the elderly was associated with several additional problems beyond the purely quantitative increases observed in the elderly. Issues of multimorbidity [54–56], polypharmacy [57, 58], frailty [59, 60], and geriatric care [61, 62] likely will pose particular challenges for needs-based care and good quality of care.

## Supporting information

S1 File (XLSX)

S2 File (XLSX)

S3 File (XLSX)

S4 File (XLSX)
